# Editorial: Plant chemoecology: Integrating micro- and macrolevel approaches in regulating secondary metabolism

**DOI:** 10.3389/fpls.2022.1119152

**Published:** 2023-01-20

**Authors:** Jinya Guo, Juncai Deng, Jiang Liu

**Affiliations:** ^1^College of Life Science, Sichuan Agricultural University, Ya’an, Sichuan, China; ^2^Sichuan Engineering Research Center for Crop Strip Intercropping System/Key Laboratory of Crop Ecophysiology and Farming System in Southwest, Ministry of Agriculture, Chengdu, China

**Keywords:** chemoecology, plant, environment, secondary metabolism, metabolomics

For millions of years, with changes in ecological geography and environmental climate, various ecological elements have been interacting with each other constantly, while plants have gradually acquired the ability to adapt to changes in the environment and climate during evolution. This process involves the adaptive evolution of the biosynthesis of many secondary metabolites, which forms a variety of complex chemical interaction networks. Chemoecology refers to the application of modern biological technology and analytical chemistry methods to study macro- and microecological phenomena. The characteristics of the cross integration of contemporary chemoecology and systems biology research have been gradually highlighted, and chemoecology has entered the omics era. Today, chemoecology can be defined as an interdiscipline that uses multidimensional analytical chemistry methods to reveal the interactions among species, within species and between living environments mediated by chemical substances and then explains the principles of ecology. In this research field, micro methods (analytical chemistry: chromatography/mass spectrometry) are used to solve macro (ecology: biotic/abiotic) scientific problems, while macro research strategies (systems biology: omics logic) are used to clarify the micro action mechanism (metabolic regulation: function of enzymes and genes).

The chemical ecological effects are reflected in various organizational levels of organisms, including individuals, populations, communities and ecosystems. In the past, chemoecology research focused on chemical interactions at the individual level. With the cross integration of chemoecology and other disciplines, contemporary chemoecology research involves biotic/abiotic interactions at a broader level, which is more closely related to basic disciplines such as plant molecular biology, plant physiology, genetics, and evolutionary biology (**Figure 1**). Based on the research strategies of systems biology such as metabolomics and bioinformatics, the principles of chemical ecology have also been widely used in applied disciplines such as crop cultivation and crop breeding, providing strong support for the sustainable development of modern ecological agriculture (**Figure 1**).

**Figure 1:**
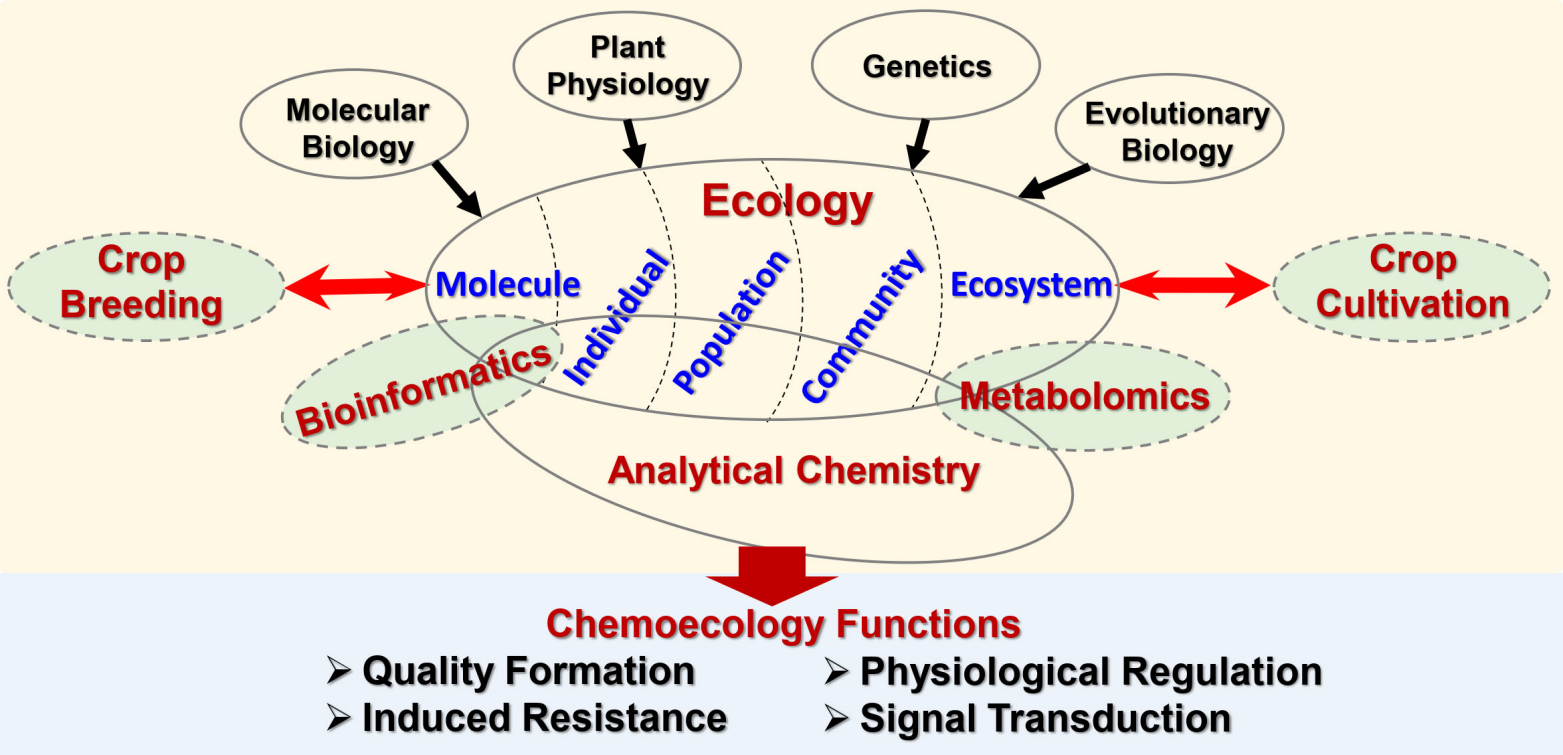
Cross amalgamation of contemporary chemoecology.

Although only 6 articles have been collected in this Research Topic thus far, it reflects several major research aspects of chemoecology. The study of the transcriptional regulation of tartary buckwheat anthocyanin synthesis and patchouli alcohol biosynthesis in *Pogostemon cablin* is a typical work at the individual level that focuses on the regulatory function of structural genes and transcription factors in the biosynthesis of natural products of individual plants. Research on the effect of drought stress on the secondary metabolism of *Glycyrrhiza uralensis* mediated by JA signaling has revealed the interaction between plants and the abiotic environment. Stress-induced metabolites are often the functional constituents in herbal medicine. Compared with the abiotic environment, the effect of the biotic environment on plant metabolism is more manual (controllable). For example, research on the regulation of rhizosphere bacteria on metabolites of *Lithospermum officinale* L. provides a new prospect for the application of microorganisms to improve the output of medicinal compounds. Moreover, genotype × environment interactions jointly determine plant secondary metabolic behavior, which has always been a global focus. The study on the accumulation characteristics of triterpenoids in different genotype subgroups of *Cyclocarya paliurus*, which were collected from different geographical locations, is a typical plant chemoecology research on the population level; this work provides important information for clarifying the environmental adaptation mechanism of plants on a larger scale. Chemoecology is closely related to plant secondary metabolism, which covers many aspects, such as chemical quality regulation, induced resistance, physiological regulation, and signal transduction (**Figure 1**). However, it must be said that the excavation of practical values is often one of the important starting points for human exploration of chemical ecology. For example, the functional development of snailase provides ideas for the efficient human use of natural flavonoids.

## Author contributions

JG and JD wrote the text, JL edited the text. All authors approve the submission.

